# Three successful births after three consecutive embryo transfers

**DOI:** 10.5935/1518-0557.20210120

**Published:** 2023

**Authors:** Claudio Barros Leal Ribeiro, Eduardo Alves Moreira, Paulo Matheus, Lúcia Helena Torres Amorim, Agostinho de Sousa Machado Júnior

**Affiliations:** 1 Universidade Federal de Pernambuco (UFPE), Recife, PE, Brazil; 2 Pernambuco Human Reproduction Center, Recife, PE, Brazil

**Keywords:** frozen embryo transfer, fresh embryo transfer, freeze-all strategy, assisted reproductive technology

## Abstract

In the last decade, frozen embryo transfer (FET) has become the preferred option for certain groups of patients rather than fresh embryo transfer. The apparent superiority of FET may be explained by improved endometrial receptivity outside stimulated cycles. In this context, our study seeks to contribute to this discussion by reporting a case involving a certain degree of originality and a success rate not commonly seen in ART. This case demonstrates that both fresh and frozen embryo transfer are good treatment options.

## INTRODUCTION

Infertility affects 15% of women of childbearing age (Bushnik *et al*., 2012). The most commonly used assisted reproductive technology (ART) procedures are in vitro fertilization (IVF) and intracytoplasmic sperm injection, or ICSI (Zegers-Hochschild *et al*., 2017). According to data from the European Society of Human Reproduction and Embryology, the clinical pregnancy (CP) rate resulting from embryo transfer was 34.5% in 2013 (European IVF-monitoring Consortium, 2017).

In this context, our study seeks to contribute to this discussion by reporting a case involving a certain degree of originality and a success rate not commonly seen in ART. 

## CASE REPORT

A 30-year-old nulligravida patient was accepted for infertility treatment. Her hysterosalpingogram revealed that her left fallopian tube was obstructed and that her right fallopian tube exhibited signs of adhesion, which rendered its topography higher than that of the right ovary. Her husband was a childless former smoker aged 47 years. His spermogram revealed a sperm density of 46,000,000 spermatozoa per milliliter, sperm morphology of 14% NF, and 30% motility. Laparoscopy was indicated, but the couple opted for IVF.

Controlled ovarian stimulation was performed using 225 IU of recombinant FSH for the first six days and 225 IU of urinary gonadotropins for the three days thereafter. On the last three days of induction, 0.25mg cetrorelix acetate was added under a short protocol. Fifteen follicles were recruited and monitored. Their sizes varied from 18 to 21 mm. LH-like exposure relied on 250µg of recombinant human chorionic gonadotropin. The oocytes were harvested 35 hours after the trigger procedure, and 12 oocytes were aspirated; ten were IVM-MII oocytes and two were IVM-MI oocytes. The mature oocytes (MII) were injected using ICSI.

**Figure 1:**
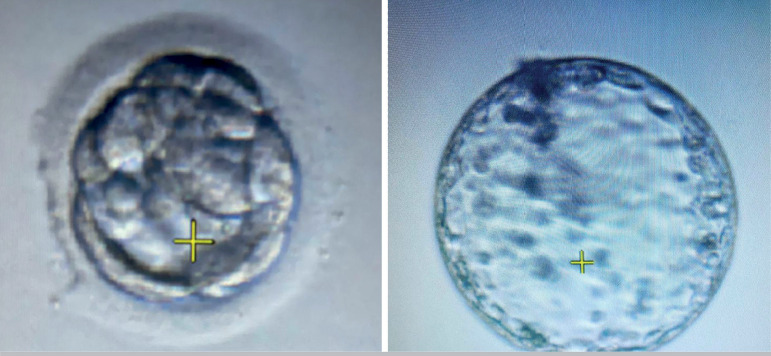
Early blastocysts.

The patients then awaited embryo development to the blastocyst stage, which occurred on the fifth day after egg fertilization. Eight blastocysts were obtained, three of which were expanded blastocysts (EB) and five were early blastocysts. Two fresh EB were transferred, while the other six were cryopreserved. The cryopreserved blastocysts were separated into two straws, one containing one EB and two early blastocysts, and the other containing three early blastocysts (Figure 1).

The two fresh blastocysts were transferred on October 5, 2013 using a Sydney IVF transfer catheter set with 30 microliters of culture medium and guided by ultrasound (US). Luteal phase support was initiated on the day of aspiration using natural micronized progesterone applied intravaginally in the form of two 200 mg gel capsules every 12 hours. Relative bed rest was recommended for the 24 hours following the transfer. After 15 days, the HCG-β result was positive, and the patient was pregnant with a single fetus. On June 16, 2014, a healthy female baby was born.

The couple returned to the clinic in 2015 to attempt a second pregnancy. A pelvic US was performed to discard endometrial or ovarian abnormalities. On the first day of the patient's next menstrual cycle, she began taking 2 mg estradiol valerate as one pill taken orally every eight hours. After eight days of use, a three-layer pattern of the endometrium was detected, with a thickness greater than 10 mm. The patient took two 200 mg vaginal natural micronized progesterone capsules every 12 hours, and the embryo transfer was scheduled for day 13 of the patient's menstrual cycle. The first straw was thawed and, of the three frozen embryos, one was categorized as not viable. Two blastocysts were transferred on September 29, 2015. After 15 days, HCG-β results were positive, and on June 9, 2016, a full-term healthy baby boy was born.

The patient's third and final transfer procedure took place on April 30, 2019. Preparatory exam results and endometrial preparation procedures were consistent with those of the second transfer. On the first day of her menstrual cycle, the patient began taking 6mg/day estradiol valerate. After 10 days, US examination revealed a three-layer pattern of the endometrium and a thickness > 10 mm. On this day, the patient was started on 800mg/day natural micronized progesterone capsules, and the embryo transfer was scheduled for day 15 of the patient's menstrual cycle. The three early blastocysts were thawed. One had developed into an EB, one had remained as an early blastocyst, and one was categorized as not viable. After 16 days, HCG-β was positive, and on January 13, 2020, a full-term healthy baby girl was born.

## DISCUSSION

The first birth following frozen embryo transfer (FET) resulted from the slow freezing technique (1984); this technique was followed by the invention of the vitrification method in 1990, a technique in which low temperatures (-196°C) are reached quickly in order to prevent the formation of ice crystals inside the cells and embryos, which would interrupt their biological activity and prevent future use, in addition to damaging the cells (Wong *et al*., 2014).

In the last decade, FET has become the preferred option for certain groups of patients rather than fresh embryo transfer. Discoveries regarding reduced endometrial receptivity associated with supraphysiological hormone levels in fresh embryo transfers have popularized the freeze-all strategy (Roque *et al* ., 2013; Labarta *et al*., 2011). Though there is sound logic and good arguments supporting the use of the freeze-all technique, its benefits cannot be generalized to the entire IVF population.

The chances of achieving a live birth (LB) were found to be consistent when the freeze-all technique was compared to fresh embryo transfer (Coates *et al*., 2017). As this case report shows, both techniques were successful at producing CP and LB in a single patient and are therefore not mutually exclusive.

## CONCLUSIONS

After the first child conceived via IVF in 1978, numerous advances in human reproduction have been made. Both fresh and frozen embryo transfer strategies should be seen as options whenever possible, even when considering a single patient. It does not seem reasonable to generalize the freeze-all strategy for use in all infertility cases.

There is a need for more and stronger data similar to the information provided in this study. Research on this topic will provide transparency, improve current understanding, and contribute to progress in science.
